# Comparative pangenomic analysis of *Campylobacter fetus* isolated from Spanish bulls and other mammalian species

**DOI:** 10.1038/s41598-024-54750-1

**Published:** 2024-02-22

**Authors:** Nerea Pena-Fernández, Medelin Ocejo, Linda van der Graaf-van Bloois, Jose Luís Lavín, Nekane Kortabarria, Esther Collantes-Fernández, Ana Hurtado, Gorka Aduriz

**Affiliations:** 1 SERIDA, Servicio Regional de Investigación y Desarrollo Agroalimentario, Carretera de Oviedo, s/n, 33300 Villaviciosa, Spain; 2 Animal Health Department, NEIKER – Basque Institute for Agricultural Research and Development, Basque Research and Technology Alliance (BRTA), Bizkaia Science and Technology Park 812L, 48160 Derio, Spain; 3 Department of Biomolecular Health Sciences, Faculty of Veterinary Medicine, Utrecht University, Utrecht, The Netherlands; 4 Department of Applied Mathematics, NEIKER - Basque Institute for Agricultural Research and Development, Basque Research and Technology Alliance (BRTA), Bizkaia Science and Technology Park 812L, 48160 Derio, Spain; 5 Animal Health Department, Faculty of Veterinary Sciences, SALUVET, Complutense University of Madrid, Ciudad Universitaria s/n, 28040 Madrid, Spain

**Keywords:** *Campylobacter fetus*, Whole-genome sequencing (WGS), Pangenome, Phylogeny, Genome-wide association study (GWAS), Gene function, Genomics, Microbial genetics

## Abstract

*Campylobacter fetus* comprises two closely related mammal-associated subspecies: *Campylobacter fetus* subsp. *fetus* (*Cff*) and *Campylobacter fetus* subsp. *venerealis* (*Cfv*). The latter causes bovine genital campylobacteriosis, a sexually-transmitted disease endemic in Spain that results in significant economic losses in the cattle industry. Here, 33 *C. fetus* Spanish isolates were whole-genome sequenced and compared with 62 publicly available *C. fetus* genomes from other countries. Genome-based taxonomic identification revealed high concordance with in silico PCR, confirming Spanish isolates as *Cff* (n = 4), *Cfv* (n = 9) and *Cfv* biovar *intermedius* (*Cfvi*, n = 20). MLST analysis assigned the Spanish isolates to 6 STs, including three novel: ST-76 and ST-77 for *Cfv* and ST-78 for *Cff*. Core genome SNP phylogenetic analysis of the 95 genomes identified multiple clusters, revealing associations at subspecies and biovar level between genomes with the same ST and separating the *Cfvi* genomes from Spain and other countries. A genome-wide association study identified *pqqL* as a *Cfv*-specific gene and a potential candidate for more accurate identification methods. Functionality analysis revealed variations in the accessory genome of *C. fetus* subspecies and biovars that deserve further studies. These results provide valuable information about the regional variants of *C. fetus* present in Spain and the genetic diversity and predicted functionality of the different subspecies.

## Introduction

*Campylobacter fetus* comprises three subspecies. Two of them, *Campylobacter fetus* subsp*. fetus* (*Cff*) and *Campylobacter fetus* subsp. *venerealis* (*Cfv*), are highly relevant veterinary pathogens commonly associated with mammals. The third one, *Campylobacter fetus* subsp. *testudium* (*Cft*), is mainly associated with reptiles and shows a clear genetic divergence from ruminant-associated *C. fetus* subspecies^1–3^. *Cff* can be found in the gastrointestinal tract of healthy ruminants, and by translocation across the intestinal mucosa, it can reach the bloodstream and colonise the gravid uterus causing abortions in sheep and sporadic abortions in cattle. *Cff*-caused sepsis and/or gastrointestinal disease have also been reported in immunocompromised humans^4–6^. *Cfv* is the causative agent of bovine genital campylobacteriosis (BGC), a bovine sexually transmitted disease distributed worldwide, and endemic in countries where natural breeding is used and cattle are extensively managed. BGC is included in the list of notifiable transmissible diseases of the World Organisation for Animal Health (WOAH, formerly known as Office International des Epizooties—OIE) and causes infertility, early embryonic death and abortions, resulting in significant economic losses for the beef cattle sector^7,8^.

*Cff* and *Cfv* are closely related phenotypically and genotypically thus hampering their identification in the laboratory. The diagnostic techniques recommended by the WOAH are culture followed by biochemical tests, the most relevant ones being the tolerance test to 1% glycine and the hydrogen sulfide (H_2_S) production test, both with positive result for *Cff* and negative for *Cfv*^8^. However, the *Cfv* biovar *intermedius* (*Cfvi*) shows intermediate results between *Cff* and *Cfv* (intolerance to 1% glycine as *Cfv* and ability to produce H_2_S like *Cff*), which can lead to misidentifications^1,9,10^. Nonetheless, recent research^11^ has reported poor reproducibility of phenotypic tests and discrepancies with genotypic tests, which challenges the use of current biochemical methods for subspecies identification. Although several PCR methods have been described for the diagnosis of *C. fetus* subspecies, the most accurate are those targeting *C. fetus*-specific *nahE* and the *Cfv*-specific *ISCfe1* insertion element^12,13^. Differentiation between *Cfv* and *Cfvi* is also possible by PCR targeting the L-cysteine transporter (L-Cyst) related to H_2_S production^14^. Other molecular methods such as matrix-assisted laser desorption ionisation-time-of-flight (MALDI-TOF), pulsed-field gel electrophoresis (PFGE) or amplified fragment length polymorphism (AFLP) have also been described, but none of these molecular tests can reliably identify *C. fetus* subspecies^15–17^. Analyses based on multilocus sequence typing (MLST) showed that mammal-associated *C. fetus* subspecies were generally separated into two large genetically homogeneous clusters (cluster A, that includes bovine derived *Cfv/Cfvi* strains belonging to sequence type (ST) 4*,* and cluster B, that encompasses strains of different STs, mostly human derived *Cff*) with an apparently clonal population structure^18,19^. However, recent studies showed that correlating MLST STs with subspecies and biovar identity was not possible due to the presence of human *Cff* strains belonging to the bovine-associated ST-4 cluster^11,20,21^. Whole genome-based studies revealed highly syntenic genomes in *Cff* and *Cfv* with 92.9% sequence identity^11,22^ alongside the presence of some gene regions exclusive in each subspecies resulting from evolutionary processes of adaptations to different hosts^22^.

A recent study revealed that BGC is endemic in Spain and widely distributed in Spanish cattle herds (especially in dehesa system herds) with a prevalence of 12.2% and 7.7% in herds and bulls, respectively^23^. In addition, this study also identified some specific mountain areas in Spain with a higher risk of BGC, where the use of communal pastures and bulls are frequent^23^. Considering these observations, it is important to investigate which subspecies of *C. fetus* are present in Spanish cattle and describe their genetic characteristics. For this purpose, complete genomes of *C. fetus* field isolates from Spanish cattle were sequenced and analysed with the following objectives: i) to taxonomically classify the isolates at subspecies level and, in the case of *Cfv*, at biovar level, ii) to study their genetic diversity by MLST typing, and iii) to perform comparative genomics of *C. fetus* field isolates with existing mammalian *C. fetus* genomes from different countries available in GenBank.

## Results

### Taxonomic identification and assignation of MLST types (ST)

The taxonomic analysis with Kraken identified four of the Spanish isolates as *Cff* and 29 as *Cfv*; this assignation was in complete agreement (100%) with the in silico PCR1. Differentiation of *Cfv* isolates at the biovar level based on L-Cyst PCR analysis (in silico PCR2) showed that 20 of the 29 *Cfv* isolates were *Cfvi* and 9 were *Cfv* (Table S1). Similarly, the 62 *C. fetus* genomes extracted from GenBank were taxonomically classified as follows: 27 *Cff*, 13 *Cfv,* and 22 *Cfvi*.

The average size of the *C. fetus* genomes analysed was 1.87 Mb (ranging from 1.73 to 2.19 Mb), with a mean G + C content of 33.1% (ranging from 32.9 to 33.4%). The mean number of genes per genome was 1,904, with *Cfvi* isolates having the highest average number of genes (1,962), followed by *Cfv* (1,946) and *Cff* (1,795) (Table S1).

In silico MLST analysis assigned the 33 Spanish genomes to 6 STs, including three novel types (Table S1). Thus, 20 *Cfvi* and 1 *Cfv* were assigned to ST-4, 2 *Cff* to ST-6 and 1 *Cff* to ST-3. The remaining 9 genomes showed novel STs: 5 *Cfv* were assigned to ST-76, 3 *Cfv* to ST-77, and 1 *Cff* to ST-78. ST-76 (strains *Cfv002*, *Cfv007*, *Cfv008 Cfv009* and *Cfv022*) was the result of a novel *aspA* allele resulting from two nucleotide transitions at positions 421 and 423 (G-A and A-G, respectively); ST-77 (strains *Cfv012*, *Cfv015* and *Cfv020*) carried a G-A nucleotide change at position 421 of the *aspA* gene; and, ST-78 (strain *Cff004*) had a nucleotide substitution of a thymine for a cytosine at position 126 of the *glnA* gene (Table S2). Most of the *Cfv* and *Cfvi* genomes downloaded from GenBank (31/35) belonged to ST-4, while the *Cff* genomes belonged to 8 different STs.

### Pangenome analysis of mammal-associated *C. fetus* strains

**Figure 1:**
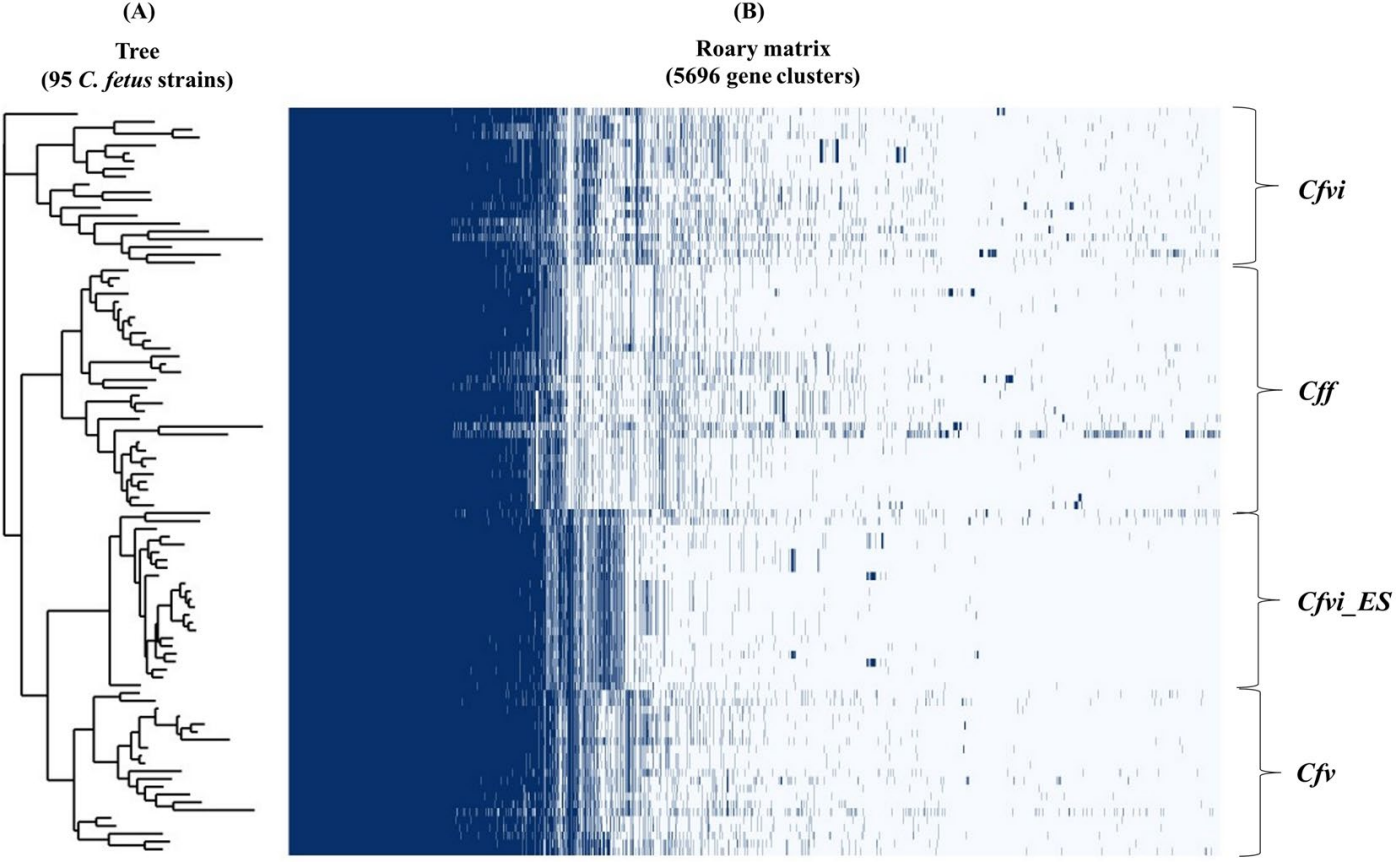
Pangenome analysis of 95 mammal-associated *C. fetus* genomes. (**A**) Dendrogram representing the grouping of the 95 *C. fetus* genomes according to the distribution of their accessory genes. (**B**) Roary matrix representing the complete genetic profile of each of the genomes based on presence/absence of core and accessory genes. *Cfvi*, *C. fetus* subsp. *venerealis* biovar *intermedius* isolates from other countries; *Cfvi_ES*, *C. fetus* subsp. *venerealis* biovar *intermedius* from Spain; *Cff*, *C. fetus* subsp. *fetus*; *Cfv*, *C. fetus* subsp. *venerealis*.

The analysis of the 95 ruminant-associated *C. fetus* genomes showed a total of 5696 genes including 1365 core genes (999 core genes and 366 soft-core genes) and 4331 accessory genes (948 shell genes and 3383 cloud genes). The genes presence/absence matrix revealed distinct patterns among the different taxonomic groups (Fig. 1B), which were supported by the PCA analysis (Figure S1) that showed a clear clustering of the genomes by subspecies (PERMANOVA, *p*_*adj*_ = 0.001). Differences were also observed in the genetic profile of the Spanish *Cfvi* genomes compared to the rest of the genomes, while the *Cfvi* genomes from other countries showed a considerable heterogeneity, showing a more dispersed clustering (Figs. 1 and S1).

**Figure 2:**
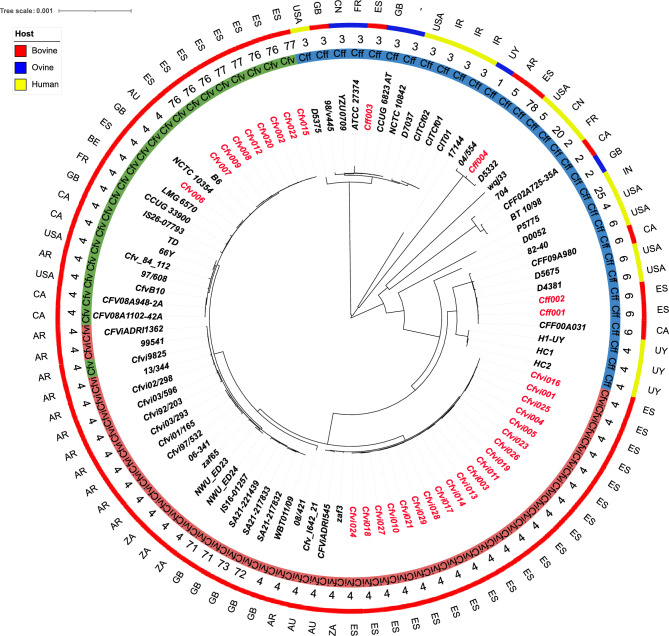
Global phylogeny of *Campylobacter fetus* based on core genome SNP analysis of 95 *C. fetus* genomes. First (outer) ring represents country of origin of the strains. Second ring represents the isolation source of strains (red, bovine; yellow, humans; blue, ovine). Numbers in the third ring represent the MLST sequence type (ST). The fourth ring (coloured lines) represents the taxonomic assignation at the subspecies level (*Cfv*—*C. fetus* subsp. *venerealis*, green; *Cfvi*—*C. fetus* subsp. *venerealis* biovar *intermedius*, red; *Cff*—*C. fetus* subsp. *fetus*, blue). Isolates sequenced in this study are labelled in red.

The phylogenetic analysis based on the core genome SNPs (1365 genes) showed multiple clusters (Fig. 2). The genomes taxonomically classified as *Cff*, originating from different host species (cattle, sheep, and human) and countries, displayed notable diversity as evidenced by the wide range of different STs, exceeding the diversity observed in *Cfv* and *Cfvi*. Nevertheless, despite this diversity, the *Cff* genomes consistently clustered together due to their shared STs. The *Cfv* (including *Cfvi*) genomes were all isolated from cattle and showed more similar core genomes. However, two subclusters were observed. The first one included all the Spanish *Cfvi* genomes (all of ST-4), and three ST-4 *Cfvi* genomes from Australia and South Africa. The remaining *Cfv* and *Cfvi* genomes formed a separate subcluster in which the genomes were separated at the biovar level into clearly different branches. The non-Spanish *Cfvi* genomes and *Cfvi9825* (taxonomically classified as *Cfv*) clustered together regardless of their ST (Fig. 2)*.* Although genomes taxonomically classified as *Cfv* clustered together, those originating from countries other than Spain (all assigned to ST-4) showed greater variability compared to the Spanish genomes, which were assigned to new STs (ST-76 and ST-77) and formed a separate cohesive subgroup. Phylogenetic analysis also revealed certain geographical association for *Cfv* and *Cfvi* genomes. Thus, *Cfvi* genomes from the same countries of origin clustered on the same branch, and for *Cfv* genomes, those from America were more similar to each other, while the European genomes were grouped separately. No clear geographic relations were observed for the *Cff* genomes.

Analysis of the accessory genome, like the core genome analysis, showed clear clustering of the genomes at the subspecies level. In this case, when presence/absence of accessory genes was compared, four different groups were identified (Fig. 1A), as the *Cff* genomes clustered on a single subcluster of the dendrogram. On the other hand, in contrast to the analysis of the core genomes, the *Cfvi* Spanish genomes shared a higher number of accessory genes with the *Cfv* genomes, while *Cfvi* genomes from other countries clustered in a separate subcluster.

GWAS analysis identified a set of genes specific for each subspecies and the biovar *intermedius* (Tables S3–S5). A total of 180 genes strongly associated with *Cff* were identified, 66 of which were exclusively found in *Cff* genomes albeit not in all of them (n = 31). Of the 233 genes strongly associated with *Cfvi*, 68 were found only in biovar *intermedius* genomes but were not present in all *Cfvi* genomes (n = 42). On the other hand, 157 genes strongly associated with *Cfv* were detected, and the *pqqL* gene, which codes for a zinc-dependent peptidase of the M16 family, was present in all genomes of this subspecies (n = 22). When these analyses were performed separating the Spanish genomes from the genomes of other countries, no specific genes associated to the Spanish genomes were found for any of the *C. fetus* subspecies or biovar.

### Functional characterization of genes comprising the different pangenome categories

To determine the potential functionality associated with the genes belonging to the different pangenome categories (core and accessory), we performed a functional classification based on the KEGG database. Of the 5696 genes that comprised the pangenome, a total of 1746 genes could be annotated, 907 from the core genome (679 core, 228 soft-core) and 839 from the accessory genome (314 shell and 525 cloud genes). Most of the annotated core genes were related to genetic information processing (10.9%), signalling and cellular processes (10.4%) and carbohydrate metabolism (8.7%). The accessory genes were mostly related to signalling and cellular processes (16.8%), genetic information processing (11.7%) and membrane transport (11.7%). Functionality analysis also showed that most of the gene functions observed were mainly encoded by core genes and some of them, such as biodegradation and metabolism of xenobiotics, immune system, environmental adaptation, and aging, were encoded exclusively by core genes. On the other hand, processes linked to membrane transport (bacterial secretion system), replication and repair, cell communication such as quorum sensing (cell community in prokaryotes), infectious diseases and signalling and cellular processes related with cell defence, were mostly encoded by accessory genes (Figure S2).

**Figure 3:**
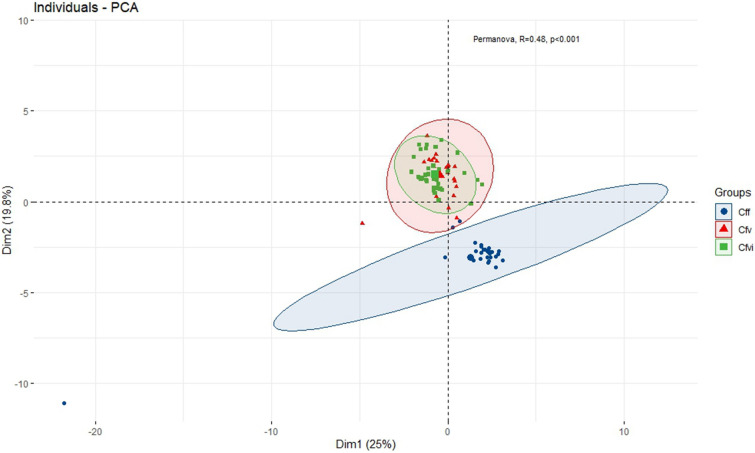
Principal component analysis based on the functional analysis of the accessory gene content of the genomes of *C. fetus* subspecies and biovar. *Cfv—C. fetus* subsp. *venerealis; Cfvi—C. fetus* subsp. *venerealis biovar intermedius; Cff—C. fetus* subsp. *fetus*.

PCA analysis based on gene functionality showed that there were significant differences between the *C. fetus* subspecies, including the *Cfvi* biovar (PERMANOVA *p*_*adj*_ = 0.001) (Fig. 3). When the same analysis was performed considering the Spanish *Cfvi* genomes as a distinct group, significant differences in the functionality of the accessory genome were observed between the Spanish *Cfvi* genomes and the rest of the *Cfvi* genomes (*p*_*adj*_ = 0.001) and the *Cff* genomes (*p*_*adj*_ = 0.001). Conversely, no significant differences in functionality were observed between *Cfv* genomes and Spanish *Cfvi* genomes (*p*_*adj*_ = 0.079). These results are consistent with those obtained in the study of the accessory genome composition where these two groups of genomes grouped in the same subcluster (Fig. 1A).

Overall, the proportion of genes encoding the different functions observed was significantly higher in the *Cff* genomes group (*p*_*adj*_ < 0.001). In contrast, *Cfv* and biovar *intermedius* had a significantly higher proportion of genes related to membrane transport (*p*_*adj*_ < 0.004), genetic information processing (*p*_*adj*_ < 0.05), and communication, such as quorum sensing functions (cell community in prokaryotes) (*p*_*adj*_ < 0.05) which represented a significantly higher proportion in the *Cfv* and biovar *intermedius* groups. The functions related to processes involved in bacterial infectious diseases were proportionally more represented in the Spanish *Cfvi* genomes (*p*_*adj*_ < 0.001) and included three alleles of the cytolethal distending toxin B subunit gene (*cdtB*), a homologue of the *hp0169* gene of *Helicobacter pylori* which encodes a putative collagenase (group 1741, prokka classification), two alleles of the *phaC* gene of *Bordetella pertussis* (groups 289 and 292), and the *lepA* gene involved in the process of host cell invasion in *Legionella pneumophila*. Finally, functions related to transcription, xenobiotic degradation and metabolism, and the metabolism of other amino acids did not show significant differences in their distribution across subspecies (Figure S3).

## Discussion

Several studies have demonstrated that WGS is an efficient method to accurately characterize *C. fetus* subspecies and to investigate their genomic differences, valuable for the development of new and more accurate diagnostic tools^5,24,25^. Under this premise, in the current investigation, the genomes of 33 Spanish *C. fetus* isolates were sequenced and compared with other publicly available genomes of mammalian-associated *C. fetus* from other countries. Taxonomic identification by in silico PCR^13^ and Kraken^26^ showed perfect agreement for the identification of *Cff* and *Cfv*. These results were consistent with the PCA and phylogenetic analysis, which clustered the genomes at subspecies level. As previously reported^27–29^ and confirmed in this study, the taxonomic results provided by Kraken are consistent with commonly used reference methods for the identification of bacterial genomes, making it a useful and precise classification tool.

Previous studies showed the close genetic relatedness and low genomic and ST variability among mammal-associated *C. fetus* subspecies and recommended the use of MLST for long-term epidemiological and phylogenetic analyses^13,18^. In this study, MLST separated the 95 *C. fetus* genomes into 14 different STs, most of them (9/14) associated to *Cff* isolated from different hosts. *Cfv/Cfvi* isolates (all from cattle) were mostly assigned to ST-4, a sequence type associated with the bovine lineage of *Cfv/Cfvi* isolates ^11,18^ and only sporadically detected in *Cff* isolates from humans^19,20^. Additionally, two new STs (ST-76 and ST-77) were described in Spanish *Cfv*. Until this study, STs other than ST-4 had only been described for *Cfvi*^18,30,31^ and *Cfv* had been characterized as a genetically homogeneous species exclusively linked to ST-4. These results indicate a greater genetic diversity among *Cfv* isolates from Spain.

Core genome based phylogenetic analysis clustered the genomes at the subspecies level; the only exception was one genome (*Cfvi*9825) that grouped together with *Cfvi* genomes despite being taxonomically identified as *Cfv*. This genome has previously exhibited discordant biochemical classifications in various labs^18,32^. Consistent with another investigation^11^, a remarkable genetic similarity was found between strains sharing the same ST as they clustered together. Also, in agreement with the MLST analysis, phylogenetic analysis showed that the *Cff* genome group exhibited a higher genetic variability compared to the *Cfv* and *Cfvi* groups, which presented more homogeneous core genomes. This homogeneity may be attributed to the restrictive adaptation of *Cfv* and its biovar *intermedius* to a particular host^18,21^. Although no significant differences between *Cfv* and *Cfvi* genomes were found in other studies^11,21^, our phylogenomic analysis revealed a clear separation of *Cfv* genomes at the biovar level. In addition, it was also observed that the *Cfvi* genomes from Spain showed a clonal structure and clustered separately from the rest of the *Cfv/Cfvi* genomes, suggesting the presence of a regional *Cfvi* variant circulating in Spain, similar to what has been reported in Argentina or Germany^25,33^. The situation was similar for the Spanish *Cfv* strains, which albeit being more closely related to *Cfv* from other countries, clustered separately. The fact that the Spanish *Cfvi* genomes shared a greater number of accessory genes with the *Cfv* genomes could indicate shared adaptive strategies or common functional traits.

Several PCR diagnostic methods are currently available to differentiate between mammal-associated *C. fetus* subspecies, but none of them are 100% accurate^13,33^. Therefore, the development of new and more reliable methods is crucial. GWAS has proven useful for the detection of new genetic targets associated with a trait of interest in bacterial populations^34^. In this study, a considerable number of genes strongly associated with each subspecies were identified, however in the case of *Cff* and *Cfvi* none of these genes were found to be present in all strains of each group. Only for *Cfv* a single gene (*pqqL*) was detected in all strains while being absent from *Cff* and *Cfvi*. The *pqqL* gene encodes a zinc-dependent peptidase, which in *E. coli* has been implicated in host adaptive functions in systemic infection processes in mammals^35,36^. Its presence in all *Cfv* genomes suggests that it might play an important role in the biology of this subspecies. Further investigations on the function of this gene could provide information on its contribution to *Cfv* survival, adaptation, or pathogenicity. In addition, this new marker could open a door to the development of more efficient *Cfv* typing methods at the biovar level, replacing current procedures (such as H_2_S production test and conventional PCR)^8,32^. However, a larger number of *C. fetus* genomes needs to be analysed to validate our observations.

The functional analysis of the *C. fetus* genomes showed that, similar to other *Campylobacter* species, the core genome of *C. fetus* is enriched in genes encoding fundamental processes for cell survival^37,38^. Compared to the core genome, the accessory genome usually encodes functions related to pathogenicity and adaptation to different environments or hosts and is not usually involved in functions essential for the survival of microorganisms^38^. In our study, we observed that the most abundant accessory genes encode mechanisms of cellular defence and adaptation to different environments. The subspecies of *C. fetus* are ecologically distinct, as *Cfv* is a pathogen restricted to the genital tract of cattle while *Cff* is considered an intestinal pathogen and can invade a wide variety of hosts^4–7^.

Our analysis of accessory genome functionality by groups showed significant differences between *C. fetus* subspecies and biovar with *Cfv* and *Cfvi* having a higher proportion of genes involved in quorum sensing and membrane transport as secretion systems compared to *Cff*. In other bacterial species such as *Bacillus*, quorum sensing has been shown to control gene expression synchronously in bacterial clusters during biofilm formation and virulence factor secretion^39^. Secretion systems facilitate the transfer of genetic material between bacteria; particularly in *Cfv*, the Type IV secretion system is involved in membrane transport functions and in the horizontal gene transfer^40^. It was also observed that the Spanish *Cfvi* strains presented a higher proportion of genes involved in bacterial infectious diseases processes that have been related to the ability to colonize hosts more efficiently^24,41–43^. Our study provides generic insight into the differences in accessory genome composition and functionality among mammalian-associated *C. fetus* subspecies. However, the significant proportion of unknown genes or genes with unidentified function, mainly in the accessory genome, underlines the strong need for further extensive analysis of the *C. fetus* genome. This will enable a more precise comprehension of the differences between subspecies and biovar, providing a more complete understanding of its genetic and functional diversity.

## Conclusions

Whole genome-based analysis allowed accurate identification of *C. fetus* subspecies and biovar and the assessment of their genome diversity. Taxonomic classification of Spanish *C. fetus* genomes revealed two novel MLST types (ST-76 and ST-77) for *Cfv* genomes and one novel ST (ST-78) for a *Cff* genome. Phylogenomic analyses clustered the *C. fetus* genomes at the subspecies and biovar level and separated the *Cfvi* Spanish genomes from *Cfvi* genomes from other countries, suggesting a regional variant circulating in Spain. Using GWAS, the *pqqL* gene was identified as uniquely present in all *Cfv* genomes thus being a potential candidate for the development of new and more accurate methods for the identification of *Cfv* biovars. Although significant differences in the functionality of the accessory genome of *C. fetus* subspecies were observed, further explorations are needed to determine the precise genetic and functional differences between *C. fetus* subspecies and biovar.

## Methods

### Strain selection

Thirty-three Spanish *C. fetus* isolates from bull preputial scraping (n = 31) and faeces (n = 1), and from cow vaginal mucus (n = 1) were selected for whole-genome sequencing (WGS) (Table S6); all isolates had been previously characterized in our laboratory using both phenotypic (biochemistry) and molecular methods (PCR)^44^. For genome comparative analyses, complete *C. fetus* genomes were retrieved from GenBank using the following search workflow: GenBank > Genome > *Campylobacter fetus* > Genome Assembly and Annotation Report (accessed, 15 May 2023). Only genomes from mammal-associated *C. fetus* subspecies and with scaffolds counts below 400 were considered. The quality of the GenBank genomes was then assessed with CheckM v1.1.6^45^, and those with less than 95% completeness and more than 5% contamination were discarded. Thus, a total of 62 *C. fetus* genomes from countries other than Spain met the inclusion criteria, *i.e.*, 40 from cattle, six from sheep and 16 from humans (Table S6).

### Bacterial culture, genomic DNA extraction, whole-genome sequencing and assembly

The 33 Spanish *C. fetus* isolates were obtained from the isolate collections of SALUVET, NEIKER and the Spanish Reference Laboratory for Animal Campylobacteriosis (Laboratorio Central de Veterinaria: LCV, Madrid, Spain) (Table S6). These isolates had been stored at − 80 °C and were grown on 5% sheep blood-enriched Columbia agar (COS, Biomerieux, Marcy-l’Étoile, France) at 37 °C under microaerophilic conditions (5% O_2_, 10% CO_2_ and 85% N_2_, GENbox Microaer, Biomerieux, Marcy-l’Étoile, France) for 48 h. DNA extraction was performed from single colony cultures following the protocol for gram-negative bacteria of the DNeasy Blood and tissues kit (QIAGEN, Hilden, Germany). Genomic DNA was sent to an external company for sequencing where library preparation was performed using the NEBNext Ultra™ II FS DNA Library Prep Kit (Illumina) and Illumina NovaSeq 6000 system was used to generate 2 × 150 bp paired-end reads. The quality of the raw reads was assessed using FastQC v.0.11.9^46^. Trimmomatic v.0.38^47^ was used for Illumina adapter removal and low-quality reads (reads with a quality score < 25 over a sliding window size of 15 bp, and reads with a sequence length < 125 bp) were filtered out using PRINSEQ v.0.20.4^48^. The resulting good quality reads were de novo assembled with SPAdes v.3.15.3^49,50^. The quality of the assemblies was assessed with QUAST v.5.0.2^51^ and contigs of less than 500 bp were discarded with PRINSEQ v.0.20.4^48^.

### Genome taxonomic identification and MLST profile definition

Taxonomic classification of the genomes at the subspecies level was carried out by exact alignment of k-mers with Kraken2^26^. To confirm Kraken2 identification of *Cff* and *Cfv*, the draft genomes were subjected to in silico PCR targeting the *C. fetus*-specific *nahE* gene and the *Cfv*-specific *ISCfe1* insertion element (PCR1)^13^ with the online tool Sequence Manipulation Suite (SMS) (http://www.bioinformatics.org/sms2/pcr_products.html). Further taxonomic classification of the *Cfv* genomes to biovar level (*Cfv* or *Cfvi*) was achieved by in silico PCR targeting the L-Cyst transporter region (PCR2)^32^.

To determine the MLST STs, the draft genomes were queried against the *C. fetus* PubMLST^52^ scheme using mlst v.2.19.0 (https://github.com/tseemann/mlst). New allelic variants and ST profiles derived from this study were submitted to PubMLST (http://pub-mlst.org) for new ST definition.

### Pangenome analysis

Gene prediction and annotation of the assembled genomes were performed with Prokka v1.11^53^ using a curated database of *C. fetus* genomes owned by the Department of Biomolecular Health Sciences, Faculty of Veterinary Medicine, University of Utrecht. The annotated assemblies in Gff3 format were used as input for pangenome calculation using Roary v3.13.0^54^ with a minimum percentage of identity for BlastP of 90. Pangenome gene categories were defined as follows: core genes (shared by 99–100% of the genomes studied); soft-core genes (95–99%); shell genes (15–95%); and cloud genes (0–15%). Parsnp v1.2^55^ was used to construct a phylogenetic tree based on the core genome SNPs (Single Nucleotide Polymorphisms) among the 95 *C. fetus* genomes, using genome *Cff* 82–40 as reference. The resulting tree was rooted at the midpoint with Figtree v1.4.2 and visualised with iTOL^56^. FastTree v2.1.10^57^ was used to construct a dendrogram based on the presence/absence of accessory genes. The Python roary_plots.py script included in Roary was used to illustrate the pangenome gene presence/absence matrix. A genome-wide association study (GWAS) was performed to identify the genes associated with the *C. fetus* subspecies and biovar *intermedius*. This study was performed with Scoary v1.6.16^58^ and the criteria for selecting genes included an Odds Ratio ≥ 1, a Native *p* value of ≤ 0.0075, a Benjamini-Hochberg (B-H) adjusted *p* value of ≤ 0.05 and a best pairwise score of ≤ 0.5.

The genes comprising the different pangenome categories were transcribed into proteins using Geneious Prime v2023.0.3 software (Biomatters) for functional categories analysis. The obtained transcripts were used as input for the online bioinformatics tool BlastKOALA (http://www.kegg.jp/blastkoala/)^59^, an automated KEGG annotation and mapping server for functional categorisation of the gene set. The graphical representation of the different gene clusters and their functionalities was generated via ggplot2 v.3.4.0 R-package. To assess differences in proportion of gene functions among the different *C. fetus* subspecies (four groups in total, with Spanish *Cfvi* treated as a separate group), we conducted a Generalized Linear Model (GLM) analysis in R. Subspecies was used as the predictor variable and the relative abundance of gene functions served as the response variable. Subsequently, Bonferroni correction was applied on *p-*values to account for multiple testing, and results were considered statistically significant when *p*_adj_ < 0.05.

Principal component analysis (PCA) based on the presence/absence of pangenome genes and accessory gene function was calculated with R. Firstly, prcomp function from stats package v.4.2.2 was used to calculate the principal components, and then factoextra v.1.0.7 was implemented to account for the different PCA features calculation and access. Finally, the 3D graphical representation was possible using rgl v.1.0.1. Differences in accessory gene functionality between *C. fetus* subspecies were calculated by PERMANOVA using Bonferroni correction, considering *p*_adj_ < 0.05 as statistically significant.

## Ethical approval and consent to participate

Sample collection was carried out by veterinary practitioners strictly following Spanish ethical guidelines and animal welfare regulations (Real Decreto 53/2013). The collection of this material, being considered a routine veterinary practice, did not require the approval of the Ethics Committee for Animal Experimentation. Informed oral consent was obtained from the farm owners at the time of sample collection. All methods were performed in accordance with the relevant guidelines and regulations and complied with ARRIVE guidelines^60^.

### Supplementary Information

Supplementary Figures.

Supplementary Tables.

## Data Availability

The Whole-Genome Sequencing project of *Campylobacter fetus* is deposited at GenBank under the BioProject accession number PRJNA1019261 and includes the 33 Spanish *Campylobacter fetus* genomes sequenced in this study, listed under the BioSample codes described in Table S6. Other datasets used and/or analysed during the current study are available from the corresponding author upon request.
