# Supplementary material for: Comparative pangenomic analysis of Campylobacter fetus isolated from Spanish bulls and other mammalian species
Source: Sci Rep. 2024 Feb 22;14:4347. doi: 10.1038/s41598-024-54750-1 (PMC10884003; doi:10.1038/s41598-024-54750-1)
Supplement: Supplementary file 1 — Supplementary Figures. [file 41598_2024_54750_MOESM1_ESM.pdf]

Supplementary Information file

Comparative pangenomic analysis of *Campylobacter fetus* isolated from Spanish bulls and other mammalian species.

Nerea Pena-Fernández[1,2†], Medelin Ocejo[2†], Linda van der Graaf -van Bloois[3], Jose Luís Lavín[4],

Nekane Kortabarria[2], Esther Collantes-Fernández[5], Ana Hurtado[2*], Gorka Aduriz[2].

[1]SERIDA, Servicio Regional de Investigación y Desarrollo Agroalimentario, Carretera de Oviedo, s/n, 33300 Villaviciosa, Spain.

[2]Animal Health Department, NEIKER - Basque Institute for Agricultural Research and Development, Basque Research and Technology Alliance (BRTA), Bizkaia Science and Technology Park 812L, 48160 Derio, Spain.

[3]Department of Biomolecular Health Sciences, Faculty of Veterinary Medicine, Utrecht University, Utrecht, the Netherlands.

[4]Department of Applied Mathematics, NEIKER - Basque Institute for Agricultural Research and Development, Basque Research and Technology Alliance (BRTA), Bizkaia Science and Technology Park 812L, 48160 Derio, Spain.

[5]SALUVET, Animal Health Department, Faculty of Veterinary Sciences, Complutense University of Madrid, Ciudad Universitaria s/n, 28040, Madrid, Spain.

† These authors contributed equally to this work and share first authorship

* Corresponding author: ahurtado@neiker.eus

Figure S1. 3D graphical representation of the principal component analysis based on the presence and absence of genes from 95 *C. fetus* genomes.

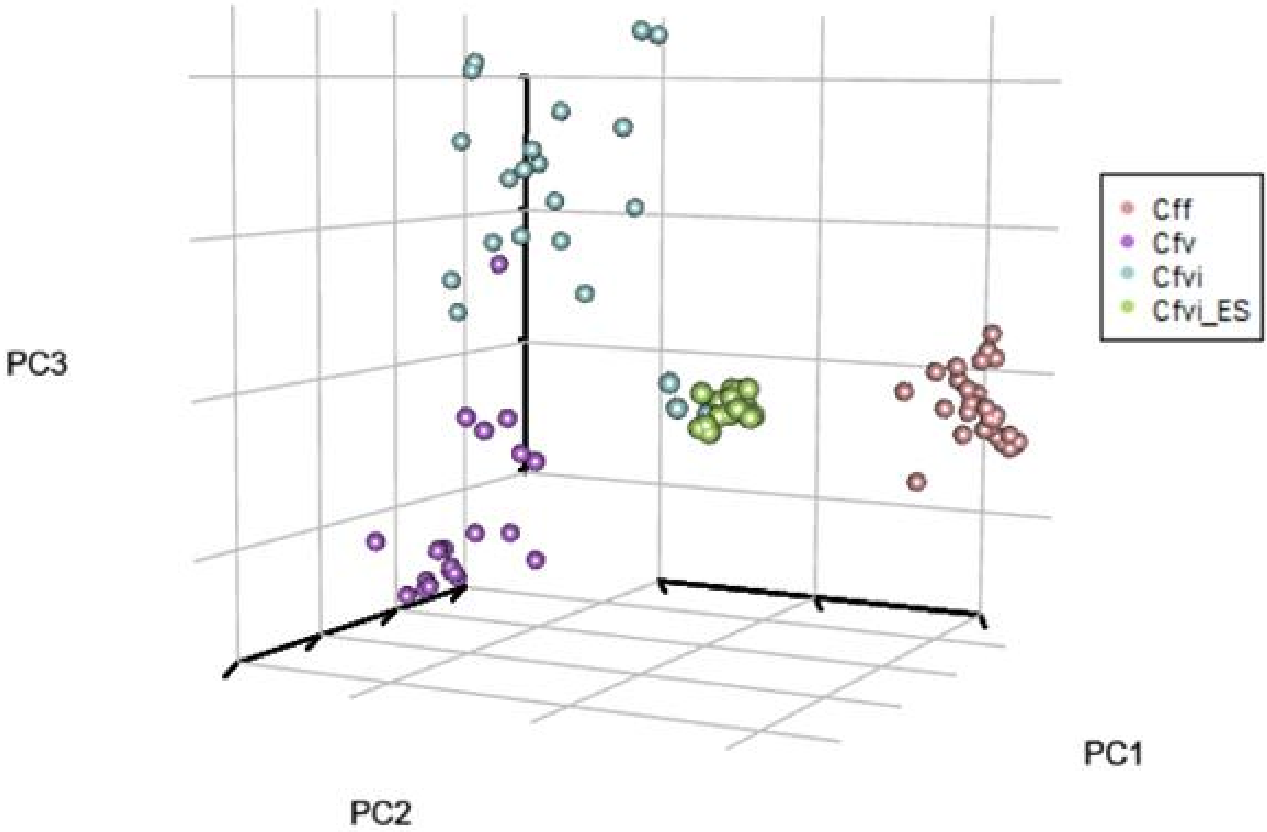

Figure S2. Bar graph showing the proportion of genes coding for each functional category in the different genome groups. Number of genes within each category and type (core and accessory) are indicated. The codes for the functional categories are those provided by KEGG orthology.

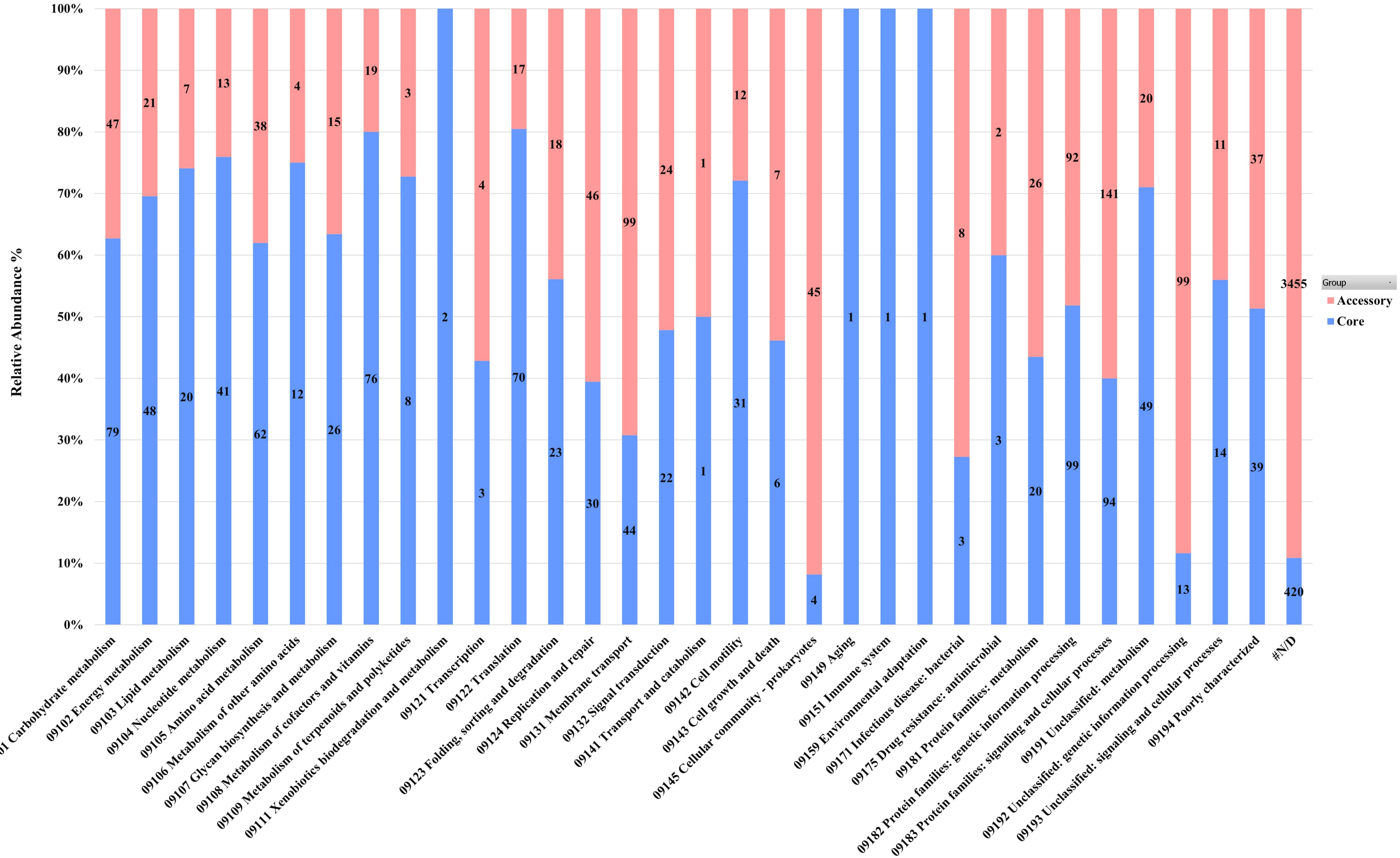

Figure S3. Boxplots showing the comparison of the proportion of genes encoding the different accessory genome functions in *C. fetus* subsp. *fetus* (*Cff*), *C. fetus* subsp. *venerealis* (*Cfv*), *C. fetus* subsp. *venerealis* biovar *intermedius* (*Cfvi*) and *Cfvi* from Spain.

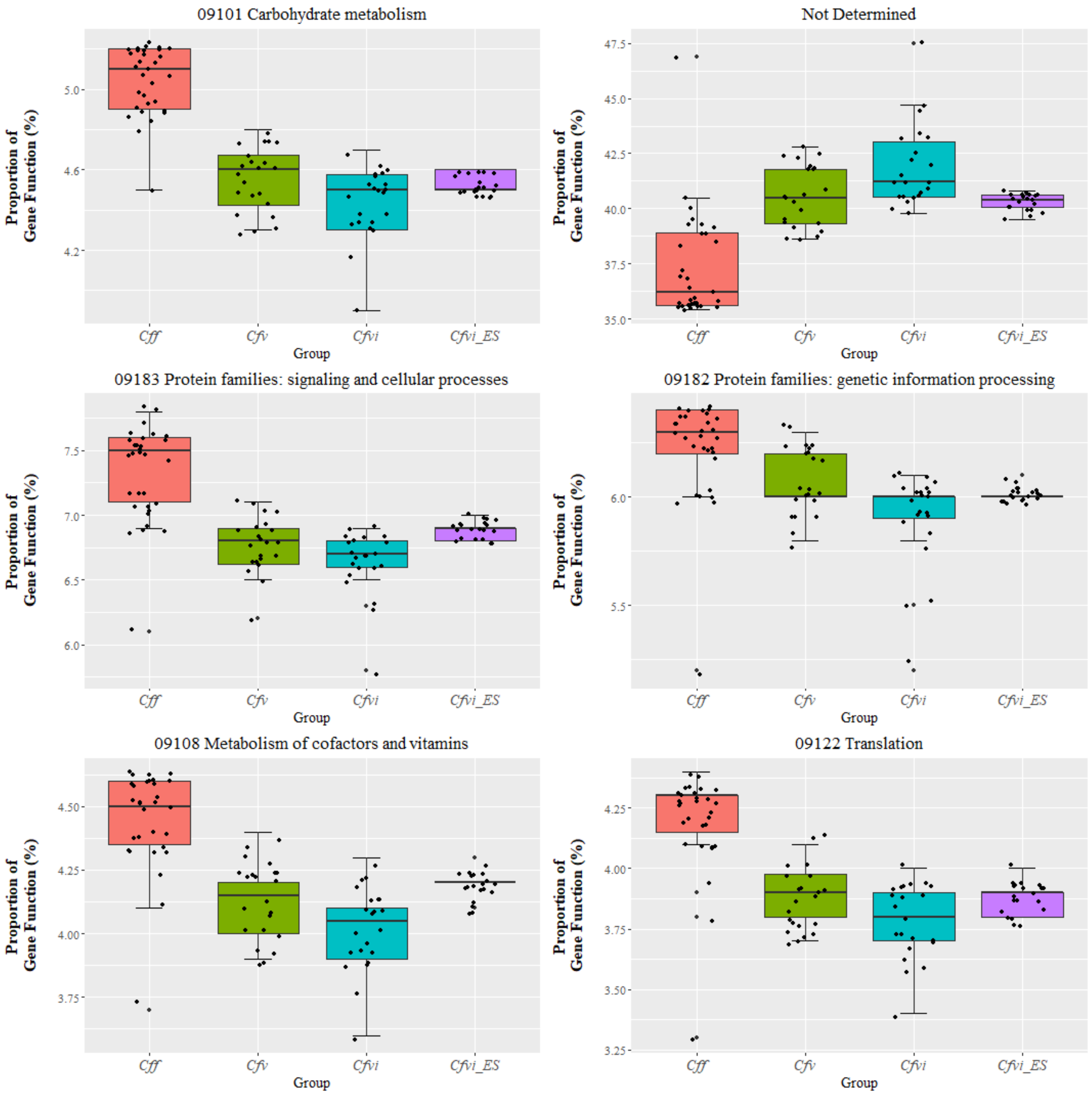

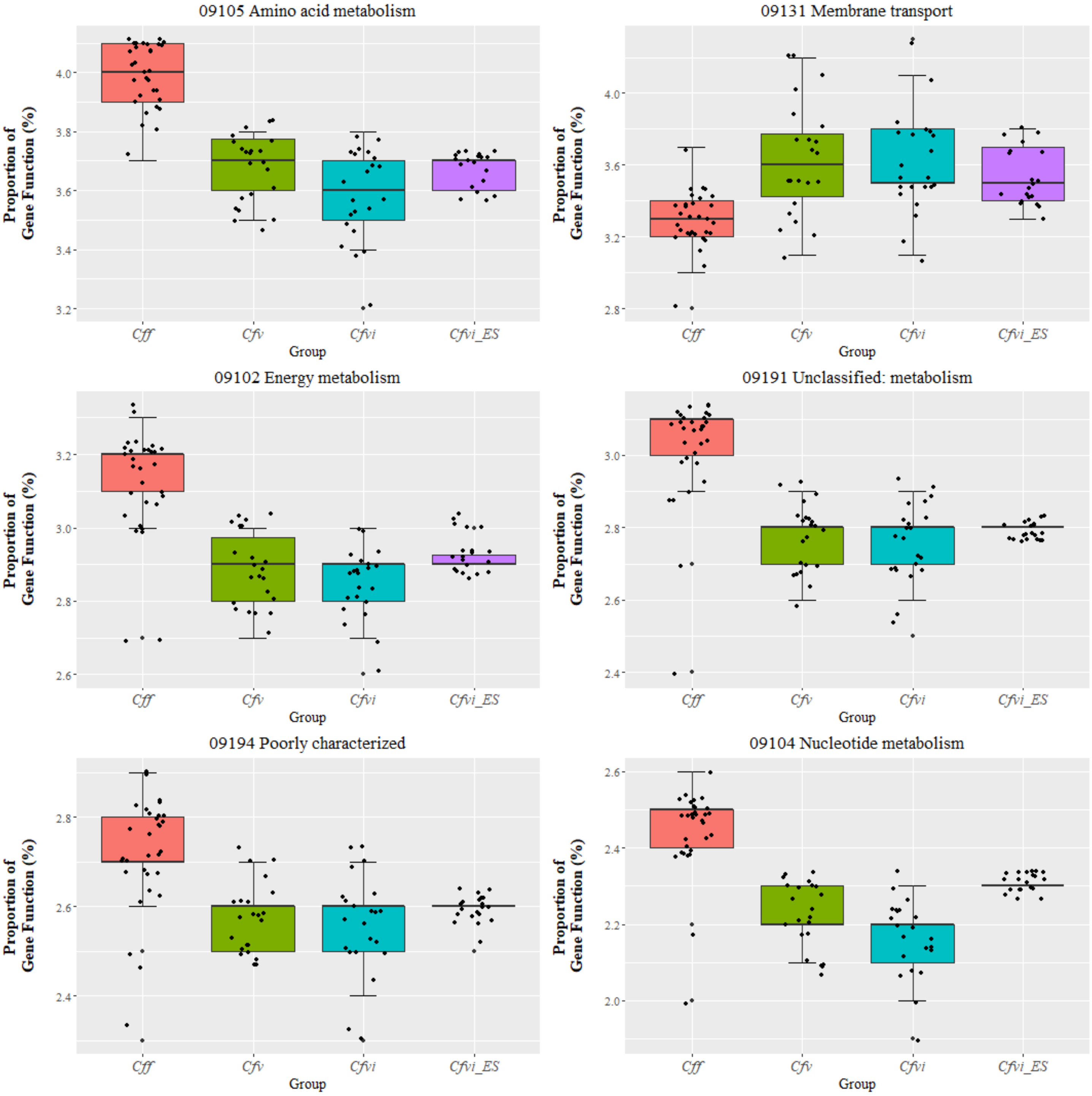
09105 Amino acid metabolism
09131 Membrane transport
09102 Energy metabolism
09191 Unclassified: metabolism
09194 Poorly characterized
09104 Nucleotide metabolism
Proportion of Gene Function (%)
Group
Cff
Cfv
Cfvi
Cfvi_ES

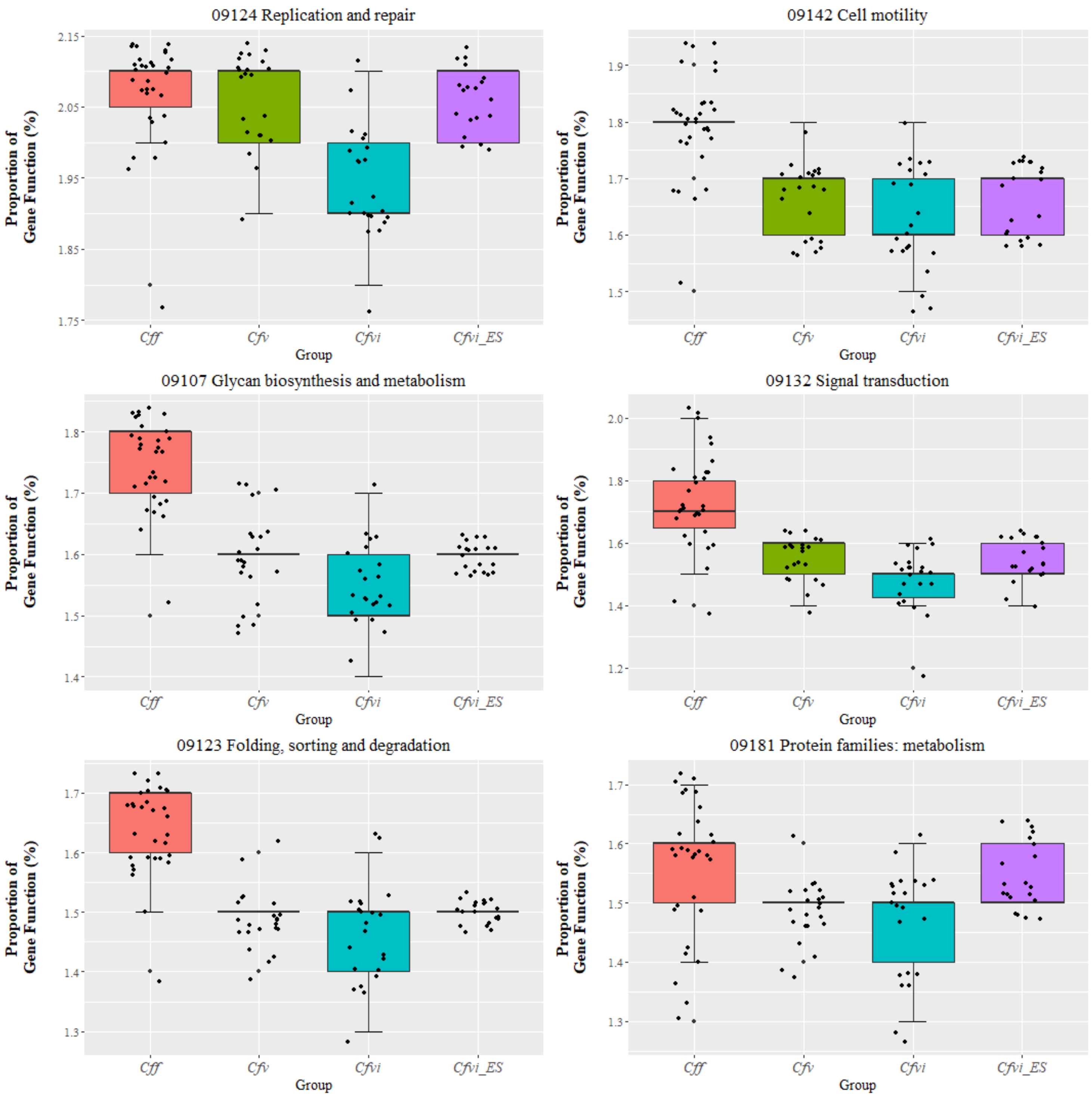
09124 Replication and repair
Proportion of Gene Function (%)
2.15
2.05
1.95
1.85
1.75
Cff
Cfv
Cfvi
Cfvi_ES
Group
09142 Cell motility
Proportion of Gene Function (%)
1.9
1.8
1.7
1.6
1.5
Cff
Cfv
Cfvi
Cfvi_ES
Group
09107 Glycan biosynthesis and metabolism
Proportion of Gene Function (%)
1.8
1.7
1.6
1.5
1.4
Cff
Cfv
Cfvi
Cfvi_ES
Group
09132 Signal transduction
Proportion of Gene Function (%)
2.0
1.8
1.6
1.4
1.2
Cff
Cfv
Cfvi
Cfvi_ES
Group
09123 Folding, sorting and degradation
Proportion of Gene Function (%)
1.7
1.6
1.5
1.4
1.3
Cff
Cfv
Cfvi
Cfvi_ES
Group
09181 Protein families: metabolism
Proportion of Gene Function (%)
1.7
1.6
1.5
1.4
1.3
Cff
Cfv
Cfvi
Cfvi_ES
Group

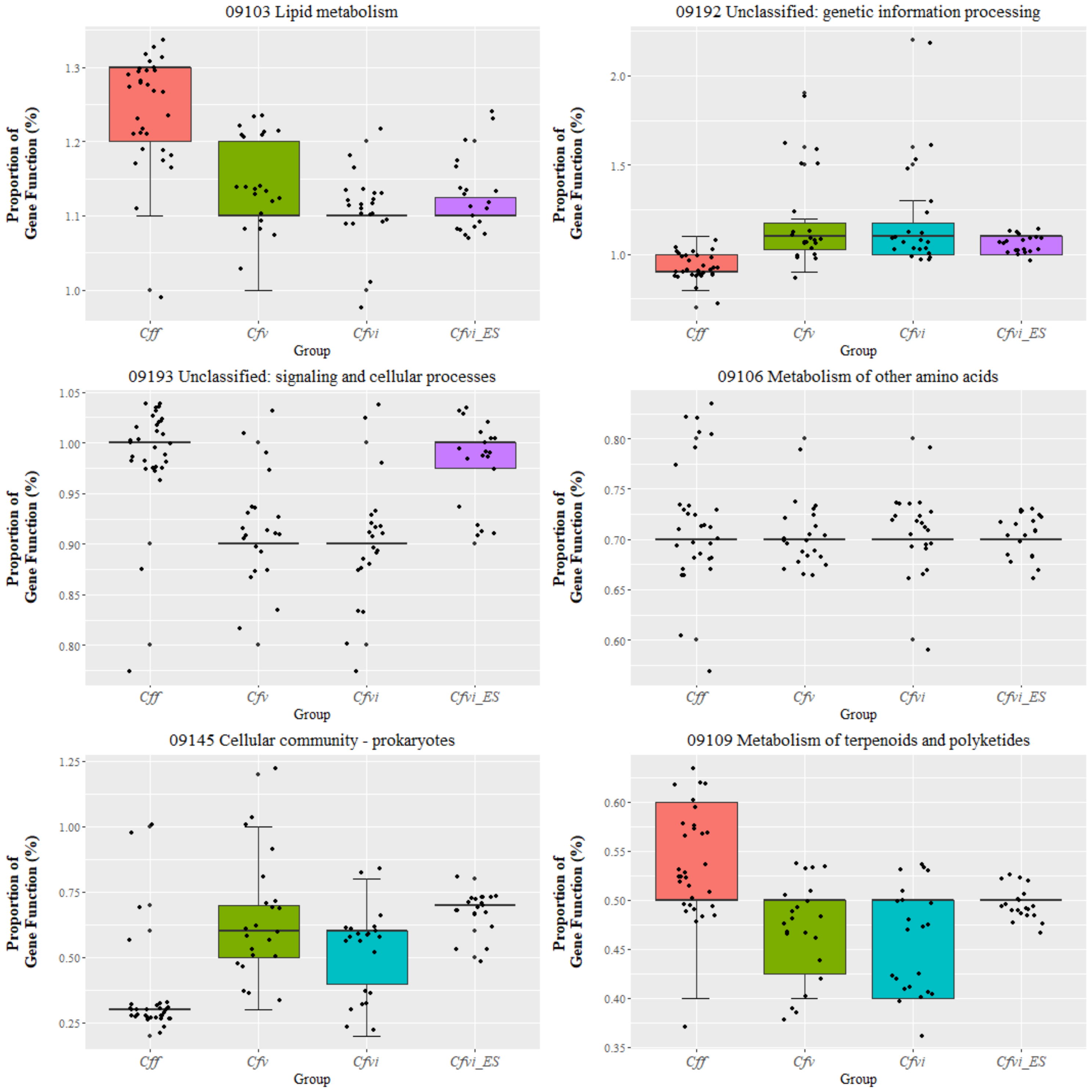
09103 Lipid metabolism
09192 Unclassified: genetic information processing
09193 Unclassified: signaling and cellular processes
09106 Metabolism of other amino acids
09145 Cellular community - prokaryotes
09109 Metabolism of terpenoids and polyketides
Proportion of Gene Function (%)
Group
Cff
Cfv
Cfvi
Cfvi_ES

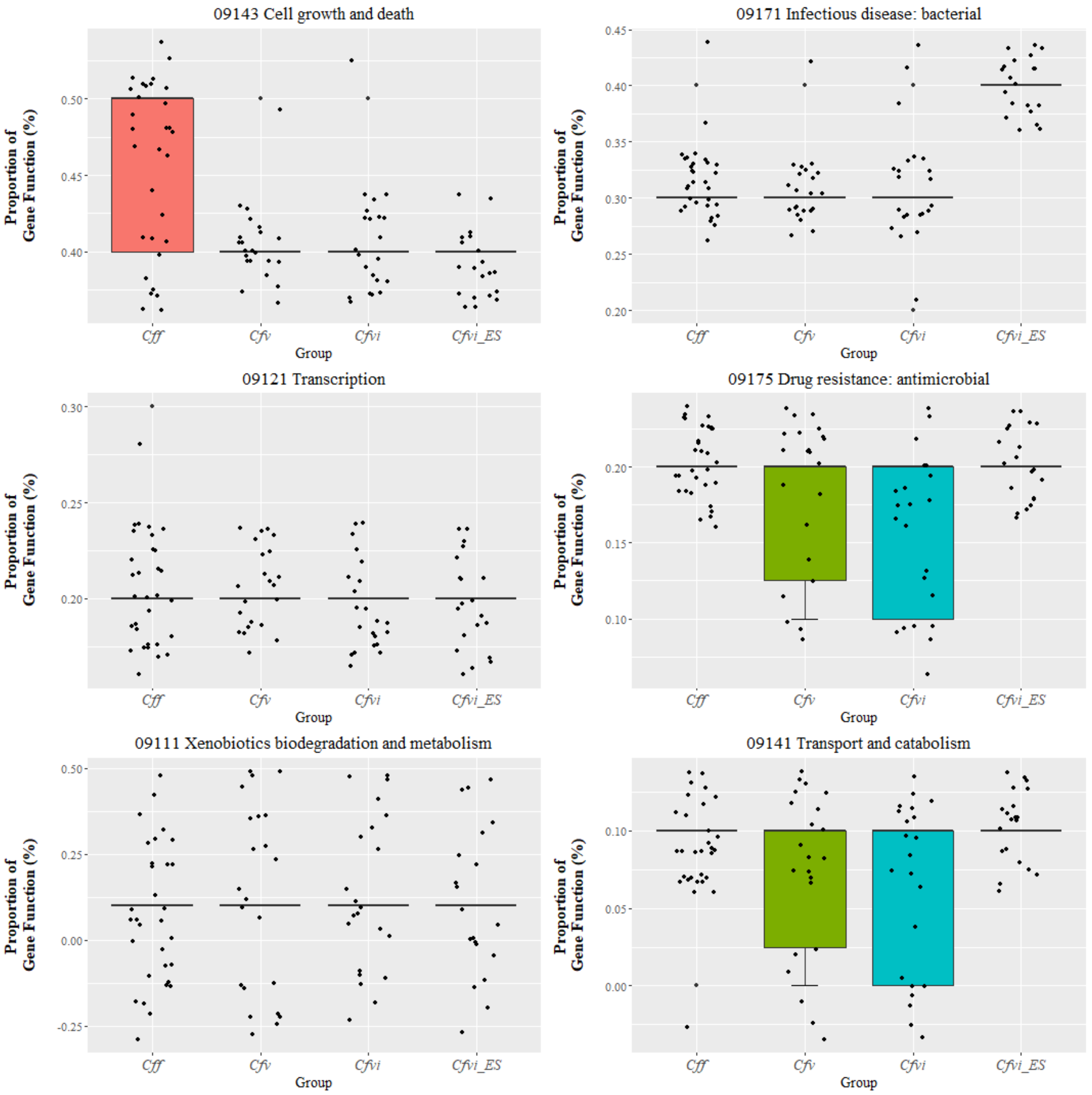

09143 Cell growth and death
Proportion of Gene Function (%)
0.50
0.45
0.40
Cff
Cfv
Cfvi
Cfvi_ES
Group
09171 Infectious disease: bacterial
0.45
0.40
0.35
0.30
0.25
0.20
09121 Transcription
0.30
0.25
0.20
09175 Drug resistance: antimicrobial
0.15
0.10
09111 Xenobiotics biodegradation and metabolism
0.50
0.25
0.00
-0.25
09141 Transport and catabolism
0.05
0.00

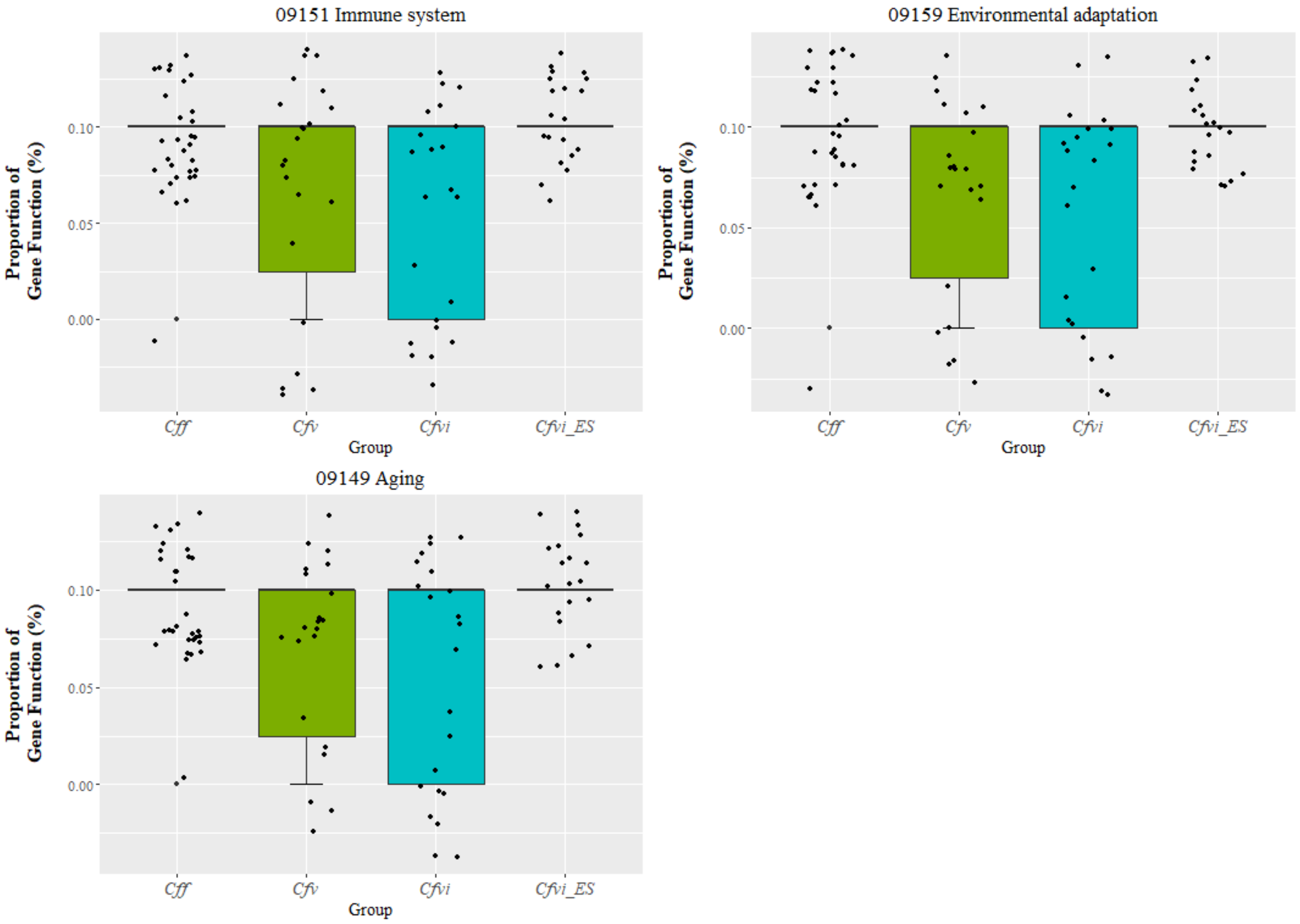
09151 Immune system
Proportion of
Gene Function (%)
0.10
0.05
0.00
Cff
Cfv
Cfvi
Cfvi_ES
Group
09159 Environmental adaptation
Proportion of
Gene Function (%)
0.10
0.05
0.00
Cff
Cfv
Cfvi
Cfvi_ES
Group
09149 Aging
Proportion of
Gene Function (%)
0.10
0.05
0.00
Cff
Cfv
Cfvi
Cfvi_ES
Group
